# 2-(Dimethyl­amino)­anthraquinone

**DOI:** 10.1107/S1600536810044636

**Published:** 2010-11-06

**Authors:** Zhuan Fei, Qun Cai, Lin Li

**Affiliations:** a Key Laboratory of Pesticide and Chemical Biology of the Ministry of Education, College of Chemistry, Central China Normal University, Wuhan 430079, People’s Republic of China

## Abstract

The mol­ecule of the title compound, C_16_H_13_NO_2_, is almost planar, with a maximum deviation of 0.013 (2) Å from the best plane; the dihedral angle between the two aromatic rings is 1.06 (1)°. In the crystal, mol­ecules are linked through weak intra­molecular C—H⋯O inter­actions, forming chains running parallel to [10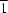].

## Related literature

For the preparation, see: Havlik *et al.* (2008 ▶). For a related structure, see: Janczak (1995 ▶).
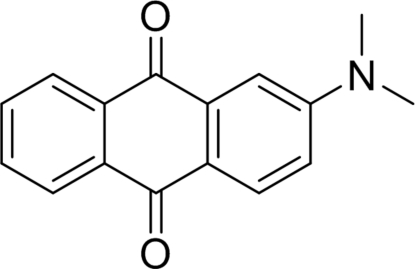


## Experimental

### 

#### Crystal data

C_16_H_13_NO_2_
*M* *_r_* = 251.27
Monoclinic, 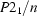

*a* = 4.8614 (6) Å
*b* = 19.945 (2) Å
*c* = 12.8624 (15) Å
β = 95.979 (2)°
*V* = 1240.3 (3) Å^3^
*Z* = 4
Mo *K*α radiation
μ = 0.09 mm^−1^
*T* = 298 K
0.23 × 0.20 × 0.12 mm

#### Data collection

Bruker SMART CCD area-detector diffractometer
14833 measured reflections
3050 independent reflections
2267 reflections with *I* > 2σ(*I*)
*R*_int_ = 0.022

#### Refinement

*R*[*F* ^2^ > 2σ(*F* ^2^)] = 0.055
*wR*(*F* ^2^) = 0.161
*S* = 1.03
3050 reflections
174 parameters
H-atom parameters constrained
Δρ_max_ = 0.30 e Å^−3^
Δρ_min_ = −0.17 e Å^−3^

### 

Data collection: *SMART* (Bruker, 1997 ▶); cell refinement: *SAINT* (Bruker, 1999 ▶); data reduction: *SAINT*; program(s) used to solve structure: *SHELXS97* (Sheldrick, 2008 ▶); program(s) used to refine structure: *SHELXL97* (Sheldrick, 2008 ▶); molecular graphics: *SHELXTL* (Sheldrick, 2008 ▶); software used to prepare material for publication: *SHELXTL*.

## Supplementary Material

Crystal structure: contains datablocks I, global. DOI: 10.1107/S1600536810044636/ng5045sup1.cif

Structure factors: contains datablocks I. DOI: 10.1107/S1600536810044636/ng5045Isup2.hkl

Additional supplementary materials:  crystallographic information; 3D view; checkCIF report

## Figures and Tables

**Table 1:** Hydrogen-bond geometry (Å, °)

*D*—H⋯*A*	*D*—H	H⋯*A*	*D*⋯*A*	*D*—H⋯*A*
C9—H9⋯O1^i^	0.93	2.50	3.272 (2)	140

Symmetry code: (i) 
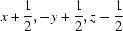
.

**Fig. 1.**
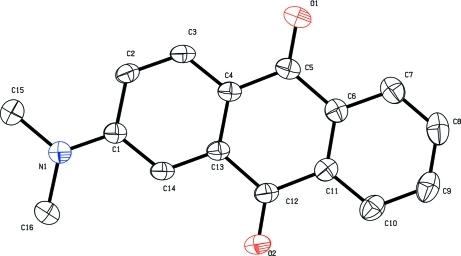
A view of (I), showing the atom-labelling scheme, with displacement ellipsoids drawn at the 30% probability level. H atoms omitted for clarity.

## supplementary crystallographic information

### Comment

The aminoanthraquinone derivatives are important compounds as dyes and intermediates.  We report here the crystal structure of the title compound.

The molecular is almost planar, with a maximum deviation of 0.013 (2)Å from the best plane. The dihedral angle between the two benzene rings is 1.06 (1)° (Fig 1). The bond distances and bond angles are in good agreement with those in a closely related crystal structure (Janczak *et al.*, 1995). In the crystal structure, the crystal packing is stabilized by a weak intramolecular C(9)—H(9)···O(1) (*x* + 1/2, -*y* + 1/2, *z* - 1/2) hydrogen bond [C(9)···O(1) 3.275 (2) Å1,Table 1].

### Experimental

The title compound was synthesized according to the reported literature (Havlik *et al.*, 2008). Crystals of (I) suitablefor X-ray diffraction were grown by slow evaporation of a chloroform-methanol(1:1) solution of the title compound under 293 K.

### Refinement

All H atoms were positioned in geometrically idealized positions and constrained to ride on their parent atoms, with C—H distances in the range 0.93–0.98 Å and *U*_iso_(H) = 1.2*U*_eq_(C) or *U*_iso_(H) = 1.5*U*_eq_(C).

### Crystal data

**Table:**

C_16_H_13_NO_2_	*F*(000) = 528
*M**_r_* = 251.27	*D*_x_ = 1.346 Mg m^−^^3^
Monoclinic, *P*2_1_/*n*	Mo *K*α radiation, λ = 0.71073 Å
Hall symbol: -P 2yn	Cell parameters from 4567 reflections
*a* = 4.8614 (6) Å	θ = 2.6–28.1°
*b* = 19.945 (2) Å	µ = 0.09 mm^−^^1^
*c* = 12.8624 (15) Å	*T* = 298 K
β = 95.979 (2)°	Block, red
*V* = 1240.3 (3) Å^3^	0.23 × 0.20 × 0.12 mm
*Z* = 4	

### Data collection

**Table:**

Bruker SMART CCD area-detector diffractometer	2267 reflections with *I* > 2σ(*I*)
Radiation source: fine-focus sealed tube	*R*_int_ = 0.022
graphite	θ_max_ = 28.3°, θ_min_ = 2.6°
phi and ω scans	*h* = −6→6
14833 measured reflections	*k* = −26→26
3050 independent reflections	*l* = −17→17

### Refinement

**Table:**

Refinement on *F*^2^	Primary atom site location: structure-invariant direct methods
Least-squares matrix: full	Secondary atom site location: difference Fourier map
*R*[*F*^2^ > 2σ(*F*^2^)] = 0.055	Hydrogen site location: inferred from neighbouring sites
*wR*(*F*^2^) = 0.161	H-atom parameters constrained
*S* = 1.03	*w* = 1/[σ^2^(*F*_o_^2^) + (0.0842*P*)^2^ + 0.2178*P*] where *P* = (*F*_o_^2^ + 2*F*_c_^2^)/3
3050 reflections	(Δ/σ)_max_ < 0.001
174 parameters	Δρ_max_ = 0.30 e Å^−^^3^
0 restraints	Δρ_min_ = −0.17 e Å^−^^3^

### Special details

**Table:**

Geometry. All e.s.d.'s (except the e.s.d. in the dihedral angle between two l.s. planes) are estimated using the full covariance matrix. The cell e.s.d.'s are taken into account individually in the estimation of e.s.d.'s in distances, angles and torsion angles; correlations between e.s.d.'s in cell parameters are only used when they are defined by crystal symmetry. An approximate (isotropic) treatment of cell e.s.d.'s is used for estimating e.s.d.'s involving l.s. planes.
Refinement. Refinement of *F*^2^ against ALL reflections. The weighted *R*-factor *wR* and goodness of fit *S* are based on *F*^2^, conventional *R*-factors *R* are based on *F*, with *F* set to zero for negative *F*^2^. The threshold expression of *F*^2^ > σ(*F*^2^) is used only for calculating *R*-factors(gt) *etc*. and is not relevant to the choice of reflections for refinement. *R*-factors based on *F*^2^ are statistically about twice as large as those based on *F*, and *R*- factors based on ALL data will be even larger.

### Fractional atomic coordinates and isotropic or equivalent isotropic displacement parameters (Å^2^)

**Table:**

	*x*	*y*	*z*	*U*_iso_*/*U*_eq_	
C1	0.6324 (3)	−0.00602 (7)	0.25988 (10)	0.0429 (3)	
C2	0.6624 (3)	0.00659 (8)	0.36860 (11)	0.0478 (3)	
H2	0.5603	−0.0184	0.4120	0.057*	
C3	0.8390 (3)	0.05496 (8)	0.41090 (10)	0.0478 (4)	
H3	0.8533	0.0623	0.4827	0.057*	
C4	0.9988 (3)	0.09367 (7)	0.34945 (10)	0.0426 (3)	
C5	1.1903 (3)	0.14348 (8)	0.39853 (11)	0.0505 (4)	
C6	1.3534 (3)	0.18380 (7)	0.32916 (12)	0.0473 (3)	
C7	1.5374 (4)	0.23222 (9)	0.37330 (14)	0.0618 (4)	
H7	1.5556	0.2395	0.4451	0.074*	
C8	1.6923 (4)	0.26926 (9)	0.30989 (17)	0.0708 (5)	
H8	1.8159	0.3012	0.3395	0.085*	
C9	1.6661 (4)	0.25959 (9)	0.20389 (17)	0.0677 (5)	
H9	1.7712	0.2850	0.1621	0.081*	
C10	1.4840 (3)	0.21210 (8)	0.15899 (14)	0.0580 (4)	
H10	1.4665	0.2057	0.0870	0.070*	
C11	1.3265 (3)	0.17382 (7)	0.22145 (11)	0.0461 (3)	
C12	1.1329 (3)	0.12237 (8)	0.17157 (11)	0.0471 (3)	
C13	0.9685 (3)	0.08246 (7)	0.24153 (10)	0.0407 (3)	
C14	0.7881 (3)	0.03404 (7)	0.19746 (10)	0.0438 (3)	
H14	0.7694	0.0279	0.1254	0.053*	
C15	0.3016 (4)	−0.09571 (9)	0.28401 (14)	0.0622 (4)	
H15A	0.4210	−0.1127	0.3422	0.093*	
H15B	0.2182	−0.1325	0.2443	0.093*	
H15C	0.1597	−0.0685	0.3092	0.093*	
C16	0.3996 (4)	−0.06205 (9)	0.10626 (13)	0.0647 (5)	
H16A	0.3266	−0.0204	0.0778	0.097*	
H16B	0.2655	−0.0970	0.0911	0.097*	
H16C	0.5656	−0.0730	0.0755	0.097*	
N1	0.4616 (3)	−0.05562 (7)	0.21809 (10)	0.0534 (3)	
O1	1.2204 (3)	0.15165 (7)	0.49310 (9)	0.0779 (4)	
O2	1.1100 (3)	0.11368 (7)	0.07767 (8)	0.0725 (4)	

### Atomic displacement parameters (Å^2^)

**Table:**

	*U*^11^	*U*^22^	*U*^33^	*U*^12^	*U*^13^	*U*^23^
C1	0.0394 (7)	0.0500 (7)	0.0395 (7)	0.0056 (6)	0.0045 (5)	0.0006 (6)
C2	0.0503 (8)	0.0574 (8)	0.0372 (7)	−0.0002 (6)	0.0118 (6)	0.0048 (6)
C3	0.0533 (8)	0.0607 (9)	0.0300 (6)	0.0038 (6)	0.0072 (5)	−0.0004 (6)
C4	0.0428 (7)	0.0500 (8)	0.0348 (7)	0.0063 (6)	0.0031 (5)	0.0003 (5)
C5	0.0528 (8)	0.0572 (8)	0.0405 (7)	0.0033 (7)	0.0007 (6)	−0.0029 (6)
C6	0.0428 (7)	0.0468 (7)	0.0511 (8)	0.0049 (6)	−0.0008 (6)	0.0015 (6)
C7	0.0619 (10)	0.0573 (9)	0.0636 (10)	−0.0020 (8)	−0.0054 (8)	−0.0038 (8)
C8	0.0616 (11)	0.0525 (9)	0.0953 (15)	−0.0101 (8)	−0.0066 (9)	0.0051 (9)
C9	0.0583 (10)	0.0566 (10)	0.0880 (14)	−0.0053 (8)	0.0064 (9)	0.0207 (9)
C10	0.0536 (9)	0.0602 (9)	0.0604 (10)	0.0034 (7)	0.0073 (7)	0.0147 (7)
C11	0.0403 (7)	0.0492 (8)	0.0486 (8)	0.0069 (6)	0.0037 (6)	0.0068 (6)
C12	0.0456 (8)	0.0590 (8)	0.0371 (7)	0.0037 (6)	0.0060 (6)	0.0040 (6)
C13	0.0389 (7)	0.0486 (7)	0.0347 (6)	0.0064 (5)	0.0043 (5)	0.0024 (5)
C14	0.0456 (7)	0.0558 (8)	0.0301 (6)	0.0039 (6)	0.0043 (5)	0.0002 (6)
C15	0.0617 (10)	0.0640 (10)	0.0616 (10)	−0.0096 (8)	0.0105 (8)	0.0028 (8)
C16	0.0747 (11)	0.0685 (10)	0.0499 (9)	−0.0116 (9)	0.0008 (8)	−0.0073 (8)
N1	0.0556 (7)	0.0605 (8)	0.0445 (7)	−0.0093 (6)	0.0065 (5)	−0.0016 (6)
O1	0.0973 (10)	0.0948 (10)	0.0405 (6)	−0.0258 (8)	0.0023 (6)	−0.0124 (6)
O2	0.0806 (8)	0.1016 (10)	0.0368 (6)	−0.0258 (7)	0.0128 (5)	−0.0003 (6)

### Geometric parameters (Å, °)

**Table:**

C1—N1	1.3651 (19)	C9—C10	1.381 (3)
C1—C14	1.4079 (19)	C9—H9	0.9300
C1—C2	1.4133 (19)	C10—C11	1.394 (2)
C2—C3	1.366 (2)	C10—H10	0.9300
C2—H2	0.9300	C11—C12	1.491 (2)
C3—C4	1.3970 (19)	C12—O2	1.2138 (17)
C3—H3	0.9300	C12—C13	1.4939 (19)
C4—C13	1.3987 (18)	C13—C14	1.385 (2)
C4—C5	1.459 (2)	C14—H14	0.9300
C5—O1	1.2208 (17)	C15—N1	1.449 (2)
C5—C6	1.490 (2)	C15—H15A	0.9600
C6—C11	1.392 (2)	C15—H15B	0.9600
C6—C7	1.396 (2)	C15—H15C	0.9600
C7—C8	1.380 (3)	C16—N1	1.444 (2)
C7—H7	0.9300	C16—H16A	0.9600
C8—C9	1.370 (3)	C16—H16B	0.9600
C8—H8	0.9300	C16—H16C	0.9600
			
N1—C1—C14	121.85 (12)	C11—C10—H10	120.0
N1—C1—C2	120.95 (13)	C6—C11—C10	119.66 (14)
C14—C1—C2	117.20 (13)	C6—C11—C12	121.08 (13)
C3—C2—C1	121.02 (13)	C10—C11—C12	119.26 (14)
C3—C2—H2	119.5	O2—C12—C11	120.97 (13)
C1—C2—H2	119.5	O2—C12—C13	121.60 (14)
C2—C3—C4	121.89 (12)	C11—C12—C13	117.43 (12)
C2—C3—H3	119.1	C14—C13—C4	120.94 (12)
C4—C3—H3	119.1	C14—C13—C12	118.73 (12)
C3—C4—C13	117.77 (13)	C4—C13—C12	120.33 (13)
C3—C4—C5	119.92 (12)	C13—C14—C1	121.15 (12)
C13—C4—C5	122.31 (13)	C13—C14—H14	119.4
O1—C5—C4	121.78 (14)	C1—C14—H14	119.4
O1—C5—C6	120.58 (14)	N1—C15—H15A	109.5
C4—C5—C6	117.64 (12)	N1—C15—H15B	109.5
C11—C6—C7	119.56 (14)	H15A—C15—H15B	109.5
C11—C6—C5	121.20 (13)	N1—C15—H15C	109.5
C7—C6—C5	119.23 (14)	H15A—C15—H15C	109.5
C8—C7—C6	119.71 (17)	H15B—C15—H15C	109.5
C8—C7—H7	120.1	N1—C16—H16A	109.5
C6—C7—H7	120.1	N1—C16—H16B	109.5
C9—C8—C7	120.86 (17)	H16A—C16—H16B	109.5
C9—C8—H8	119.6	N1—C16—H16C	109.5
C7—C8—H8	119.6	H16A—C16—H16C	109.5
C8—C9—C10	120.12 (16)	H16B—C16—H16C	109.5
C8—C9—H9	119.9	C1—N1—C16	120.78 (13)
C10—C9—H9	119.9	C1—N1—C15	120.72 (12)
C9—C10—C11	120.08 (17)	C16—N1—C15	117.68 (13)
C9—C10—H10	120.0		
			
N1—C1—C2—C3	178.16 (13)	C9—C10—C11—C6	0.1 (2)
C14—C1—C2—C3	−1.1 (2)	C9—C10—C11—C12	−179.45 (14)
C1—C2—C3—C4	−0.5 (2)	C6—C11—C12—O2	−179.89 (14)
C2—C3—C4—C13	1.3 (2)	C10—C11—C12—O2	−0.4 (2)
C2—C3—C4—C5	−178.30 (14)	C6—C11—C12—C13	0.4 (2)
C3—C4—C5—O1	1.6 (2)	C10—C11—C12—C13	179.92 (13)
C13—C4—C5—O1	−178.05 (14)	C3—C4—C13—C14	−0.7 (2)
C3—C4—C5—C6	−179.42 (12)	C5—C4—C13—C14	178.97 (13)
C13—C4—C5—C6	1.0 (2)	C3—C4—C13—C12	179.82 (12)
O1—C5—C6—C11	178.32 (15)	C5—C4—C13—C12	−0.5 (2)
C4—C5—C6—C11	−0.7 (2)	O2—C12—C13—C14	0.6 (2)
O1—C5—C6—C7	−1.4 (2)	C11—C12—C13—C14	−179.66 (12)
C4—C5—C6—C7	179.60 (13)	O2—C12—C13—C4	−179.87 (14)
C11—C6—C7—C8	−0.5 (2)	C11—C12—C13—C4	−0.14 (19)
C5—C6—C7—C8	179.16 (15)	C4—C13—C14—C1	−0.9 (2)
C6—C7—C8—C9	0.6 (3)	C12—C13—C14—C1	178.63 (12)
C7—C8—C9—C10	−0.3 (3)	N1—C1—C14—C13	−177.49 (13)
C8—C9—C10—C11	−0.1 (3)	C2—C1—C14—C13	1.7 (2)
C7—C6—C11—C10	0.2 (2)	C14—C1—N1—C16	−10.0 (2)
C5—C6—C11—C10	−179.49 (13)	C2—C1—N1—C16	170.79 (14)
C7—C6—C11—C12	179.74 (13)	C14—C1—N1—C15	−179.41 (14)
C5—C6—C11—C12	0.0 (2)	C2—C1—N1—C15	1.4 (2)

### Hydrogen-bond geometry (Å, °)

**Table:**

*D*—H···*A*	*D*—H	H···*A*	*D*···*A*	*D*—H···*A*
C9—H9···O1^i^	0.93	2.50	3.272 (2)	140

Symmetry codes: (i) *x*+1/2, −*y*+1/2, *z*−1/2.
